# Nanoparticle-based radiosensitization strategies for improving radiation therapy

**DOI:** 10.3389/fphar.2023.1145551

**Published:** 2023-02-16

**Authors:** Hongxin Shen, Hong Huang, Zhimei Jiang

**Affiliations:** ^1^ Department of Pharmacy, Evidence-Based Pharmacy Center, West China Second University Hospital, Sichuan University, Chengdu, China; ^2^ Key Laboratory of Birth Defects and Related Diseases of Women and Children, Sichuan University, Ministry of Education, Chengdu, China

**Keywords:** radiosensitizers, nanoparticle strategy, radiotherapy, reactive oxygen species, radiation dose deposit, chemical drugs, antisense oligonucleotide gene, radiation-activable property

## Abstract

Radiotherapy remains the mainstay treatment for a variety of cancer forms. However, the therapeutic efficiency of radiation is significantly limited by several aspects, including high radiation resistance caused by low reactive oxygen species concentrations and a low absorption rate of radiation by tumor tissue, inappropriate tumor cell cycle and tumor cell apoptosis, and serious radiation damage to normal cells. In recent years, nanoparticles have been widely used as radiosensitizers due to their unique physicochemical properties and multifunctionalities for potentially enhancing radiation therapy efficacy. In this study, we systematically reviewed several nanoparticle-based radiosensitization strategies for radiation therapy use, including designing nanoparticles that upregulate the levels of reactive oxygen species, designing nanoparticles that enhance the radiation dose deposit, designing chemical drug-loaded nanoparticles for enhancing cancer cell sensitivity to radiation, designing antisense oligonucleotide gene-loaded nanoparticles, and designing nanoparticles using a unique radiation-activable property. The current challenges and opportunities for nanoparticle-based radiosensitizers are also discussed.

## 1 Introduction

Along with chemotherapy and surgery, radiotherapy (RT) is used as one of the mainstream treatments for cancer. Around 50 percent of cancer patients underwent at least one dose RT in their treatment process (Delaney et al., 2005). Radiotherapy shows significant effects through transmitting particulate radiation or electromagnetic radiation, which will positively have an effect on biological tissues. The delivered particles or waves after being absorbed by tissues will generate ionization which releases electrons and subsequent free radicals, generating energy deposition within the defined capacity (Bernier et al., 2006). The generated energy creates breaks in the DNA inside tumor cells, therefore slowing down the growth and division of tumor cells, and finally causes the tumor cell death. However, the frequent occurrence of tumor cell radiation resistance caused by the enhanced DNA-repair ability in tumors is one of the prime causes of radiotherapy failure. Furthermore, the outcome of radiotherapy comprises its relatively poor ability to deliver therapeutic doses specifically to the tumors and its high probability of causing damage to the surrounding healthy tissues (Barnett et al., 2009). To improve the sensitization of radiotherapy, a few products have been developed and applied in clinical settings, namely, radiosensitizers such as irisquinone capsules and sodium glycididazole for injection. On the other hand, the combination of radiotherapy and anticancer drugs induced synergistic effects on tumor treatment (e.g., application of cisplatin and Taxol). However, the challenge is that both the radiotherapy and chemotherapy currently in use cause considerable cytotoxicity to normal tissues, and free chemotherapeutic agents have low accumulation at the tumor site. Development of new radiosensitization strategies that can increase radiotherapeutic efficacy while decreasing the side effects remains the main challenge for improving the radiotherapy outcome.

With the rapid development of nanotechnology, it has been demonstrated that rationally designed nanoparticles (NPs) show a special capability to broaden the therapeutic window of radiotherapy from different aspects, resulting in excellent radiotherapeutic efficacy (Pottier et al., 2015; Ngwa et al., 2017). First, oxygen-producing nanoparticles, such as manganese dioxide nanoparticles, can promote H_2_O_2_ disintegration to produce O_2_ which is a basic factor in ensuring the efficacy of RT (Li et al., 2018). In addition, nanoparticles have the particular features of improving drug-targeted delivery to tumor tissue by using enhanced permeability, retention effect, and active targeting strategy. High-atomic-number nanoparticles, such as hafnium oxide nanoparticles, afford high radiation attenuation capacity (Le Tourneau et al., 2018). In recent years, new types of tumor-targeting nanoparticles made from radiation–activable metals have also been developed. These nanoparticles could be activated in the inner part of tumors through external beam radiation, followed by further enhanced radiation therapy; so, they can effectively avoid side effects to normal tissues.

In the current study, we systematically reviewed various nanoparticle strategies that have been developed to improve the radiation susceptibility of tumor cells during treatment. The described strategies include designing nanoparticles that remodel the tumor microenvironment, designing nanoparticles that enhance the radiation dose deposit, designing chemical drug-loaded nanoparticles for enhancing cancer cells’ sensitivity to radiation, and designing antisense oligonucleotide gene-loaded nanoparticles. The review of research progress in this field may promote the development of new advanced nanoparticle-based radiosensitizers for increasing radiation therapy efficacy.

## 2 Nanoparticle-based radiosensitization strategies

### 2.1 Designing nanoparticles that can upregulate the levels of reactive oxygen species in tumor microenvironment

Tumor microenvironment (TME), which is the environment around a tumor mass, plays important roles in tumor progression and metastasis. Growing evidence suggests that oxygen deficiency may cause tumor cells to develop radioresistance and is considered to be one major cause of the failure of radiotherapy (Kaur et al., 2005; Brown, 2007). In addition, previous studies have shown that the intensity of hypoxia within malignant gliomas is closely related to the patient’s overall survival rate after radiotherapy (Evans et al., 2004; Siegal et al., 2016). The use of rationally designed nanoparticles could lead to higher generation of reactive oxygen species (ROS), subsequently resulting in greater cytotoxicity and genotoxicity of radiation when compared to the radiation-only group. This radiation-sensitizing effect will further contribute to sustained DNA double-strand breaks (DSBs), representing a major disruption in the integrity of a genome, switching from the G_2_/M phase into a G_1_/S cell cycle checkpoint, eventually leading to cell senescence (Grall et al., 2015). For instance, studies (Wason et al., 2013) have shown that cerium oxide nanoparticles (CONPs) can significantly improve the sensitivity of pancreatic cancer cells to radiotherapy. Specifically, CONPs pretreatment showed preferentially enhanced radiation-induced ROS generation in acidic acellular solution and human pancreatic cancer cells. In addition, in acidic surroundings, CONPs were conducive to the clearing of superoxide free radicals rather than the hydroxyl peroxide, giving rise to a build-up of the latter matter, whereas in neutral pH, they were found to eliminate both. CONP administration before radiotherapy significantly potentiated the apoptosis of tumor cells both in culture and tumors, further suppressing the idea of pancreatic tumor growth and leaving the normal tissues or host mice undamaged. In addition, through the use of three human radioresistant cancer cell lines, hydrogenated nanodiamonds (H-NDs) showed cytotoxic potential after concurrent exposure to radiation, the mechanism of an event attributed to inducing DNA damage and ROS production. The situation emerged along with decreased cell impedance, activated unlock of the G1/S, G2 cell cycle check-points, and low cell mortality in the early time period (Grall et al., 2015). Therefore, nanoparticles with the function of inducing ROS overexpression may have the potential as radiation sensitizers. Whether the ROS-induced apoptosis was the result of the co-treatment of nanoparticles and irradiations was also further investigated. The specific probe dihydroethidium (DHE) was applied to determine the level of intracellular ROS. Studies revealed that the single administration of FA@SeNPs or ^125^I seed radiation used in MCF-7 cells brought about a slight increase of the intracellular ROS level in about half an hour, and later tending downward to 113.4% and 108%, respectively, at 2 h. It is interesting that the level of ROS increased to 210.2% after half an hour and kept up at 167.4% after 2 h of culture with the combination of FA@Se nanoparticles and ^125^I seed radiation. In addition, no changes were observed in the cell cycle, and only weak apoptosis was observed in MCF-7 cells treated with single FA@SeNPs or ^125^I seed radiation. On the contrary, the consolidated treatment of the FA@SeNPs and ^125^I seed irradiation group was observed to increase the level of the sub-G1 population from 19.5% (control group) to 50.5%, and a significant apoptotic effect was observed (Yang et al., 2017). Therefore, these discoveries further confirm that combined treatment of nanoparticle-based radiosensitizers and radiation drastically increased ROS overproduction accompanied by induction of apoptosis and a G2/M arrest. Similarly, the aforementioned mechanism of radiosensitization was demonstrated on various smart nanoparticles as shown in Table 1.

**TABLE 1:** Summary of NP-based radiosensitizers to upregulate the levels of reactive oxygen species.

Reference	NP-type	Size (nm)	Radiation (radiation dose)	Cell line
Grall et al. (2015)	Hydrogenated nanodiamonds	16	γ-ray (4 Gy)	Caki-1, ZR75.1_Ku70wt_,
				ZR75.1_Ku70mut_
Wason et al. (2013)	Cerium oxide nanoparticles	5–8	Ionizing radiation (5 Gy)	Human pancreatic cancer cell line L3.6 pl
Yang et al. (2017)	Folic acid-conjugated selenium nanoparticles	192	^125^I seeds	Human breast cancer MCF-7 cells
Sayed et al. (2021)	Zinc oxide-caffeic acid nanoparticles	30	γ-ray (2Gy)	Human breast cancer MCF-7, human hepatocellular adenocarcinoma HepG2 cell lines
Shin et al. (2021)	Manganese ferrite nanoparticles	21	X-ray (6Gy)	Hepa1-6 mouse hepatoma cells
Zhou et al. (2020)	Cu2-xSe@PtSe nanoparticles functionalized with HIF-1α	20.7 ± 3.0	X-ray (6Gy)	Murine breast cancer cells (4T1 cells)
Ma et al. (2019)	Quercetin-modified metal–organic frameworks, zirconium nanoparticles	125	X-ray (8Gy)	Human lung cancer cell lines A549 and HCC827 and the human breast cancer cell line MDA-MB-231
Liu et al. (2020)	Manganese dioxide nanoparticles	126.1 ± 9.6	X-ray (5Gy)	Human non-small cell lung cancer H1299 cells and human head and neck squamous cell carcinoma SCC7 cells
Huang et al. (2014)	Folic acid-modified bovine serum albumin nanoparticles	255	X-ray (8Gy)	Hela cervical carcinoma cells, L02 human hepatic cells, A375 melanoma cells, and MCF-7 breast adenocarcinoma cells
Du et al. (2020)	Gadolinium-based nanoparticle	3	γ-ray (2,4,8,10Gy)	H1299 and A549 cell lines
Moritaa et al. (2016)	Polyacrylic acid-coated titanium dioxide with H_2_O_2_ nanoparticles	124 ± 65	X-ray (−)	—

### 2.2 Designing nanoparticles that enhance the radiation dose deposit

The application of high-atomic-number (Z) nanoparticles in radiotherapy has attracted more and more attention because calculations have proved its superior characteristics by the up-and-coming enhanced radiation dose deposit, despite being in the low-energy region only. High-Z nanoparticles improve the radiation dose deposit within tumor tissues owing to their unique properties of a high photoelectric cross-section (Z^4^/E^3^, Z atomic number, E incident energy) (Podgorsak, 2016). Consequently, the local dose increase allows the reduction of the total radiation dose delivery for normal and tumor tissues. There have been an increasing number of studies involving high-Z NPs applied as radiosensitizers, such as gold (Au), hafnium (Hf), bismuth (Bi), gadolinium (Gd), and silver (Ag) NPs (Table 2). The photon beam irradiation of NPs generates enormous short-range electrons and Auger cascades, which is the principal reason for the enhancement of the microscopic absorbed dose without a significant difference in the macroscopic absorbed dose (Lin et al., 2014). The specification of radiation dose enhancement is in accordance with the dose enhancement factor (DEF), which is scientifically defined as the ratio of the absorbed dose with and without specific materials in the medium (Haume et al., 2016). It was indicated that higher Z value materials (Z > 70) would present a greater degree of dose enhancement. Nanoparticles with medium Z values (about 40–65) might provide decreasing DEF for brachytherapy sources or may stay at a steady level for external kilovolt sources (Roeske et al., 2007).

**TABLE 2:** Summary of different types of high-Z NP-based radiosensitizers involving DEF determination.

Reference	High-Z element	NP-type	Size (nm)	Radiation (radiation dose)	Cell line	DEF value
Chattopadhyay et al. (2013)	Au	HER-2-Au NPs	30	X-ray (11Gy)	K-BR-3 breast cancer cells	1.6
Maggiorella et al. (2012)	Hf	Hafnium oxide NPs	50	Cobalt¬60 (4Gy)	Human HT1080 fibrosarcoma cell line	1.4 ± 0.06 (4Gy); 1.8 ± 0.09 (6 MV)
Rehman et al. (2021)	Ag	Silver-coated titanium dioxide NPs	49.2 ± 3.13	X-ray and cobalt-60 (2,4,3,8Gy)	Macrophage JA774 A.1 cells	1.44(8Gy, X-ray); 1.2(8Gy, cobalt-60)
Zangeneh et al. (2019)	Gd	Gd NPs	1–50	X- ray (beam energy from 25 keV to 1.25 MeV)	Rodent glioma F98 cells	1.41 ± 0.11 (1.25 MeV)
Nezhad et al. (2021)	Bi	Bi_2_O_3_ NPs	50–150 (sheet NPs); 18–25 (spherical NPs)	X-ray (50 kV)	—	3.28 ± 0.37 (sheet NPs); 2.50 ± 0.23 (spherical NPs)

In addition, the radiation dose enhancement would vary based on the nanoparticles’ size, types, concentration, radiation types, incident beam energy, etc. The published literature revealed that for the lower incident energy, the dose enhancement degree was greater. In addition, a higher degree of dose enhancement was noticed in preparations containing Au, Gd, and I, followed by Fe_2_O_3_ (Hwang et al., 2017). Furthermore, owing to the impact of dose enhancement within the tumor tissues, the influence degree of photons was reduced, which subsequently resulted in decreased dosage at the reverse side of the tumor volume. The diameter of nanoparticles was also related to DEF. Nanoparticles with a larger diameter bring about higher DEF values as follows: nanoparticles with a size of 125 nm lead to a DEF value in the range of 1.040–1.000, with a size of 100 nm resulting in a DEF value in the range of 1.036–1.000, with a size of 75 nm bring about a DEF value in the range of 1.035–1.001, with a size of 50 nm size result in a DEF value in the range of 1.032–1.001, and with a size of 25 nm result in a DEF value in the range of 1.028–1.000. For materials presenting higher dose enhancement effects, the dose enhancement factor changes were more prominent with the diameter of nanoparticles. Moreover, higher concentrations of dose enhancement preparations were promising in providing significant increases of DEF by using 100-nm nanoparticles (Hwang et al., 2017). Niladri *et al.* demonstrated the clonogenic survival of cells exposed to human epidermal growth factor receptor-2 (HER-2)-targeted gold nanoparticles (Au–T) and X-rays was apparently lower than that of cells treated by single X-radiation, which meant that in terms of DEF, it is 1.6 (Chattopadhyay et al., 2013). Laurence *et al.* observed marked radioenhancement in the HT1080 fibrosarcoma cell line by combined administration of hafnium oxide nanoparticles and irradiation. Under the conditions of the cobalt-60 source and 6-MV linear accelerator, the clonogenic surviving fraction was significantly decreased. The estimation of radiation dose enhancement was specified as DEFs at 1.4 ± 0.06 for 4 Gy and 1.8 ± 0.09 for the 6-MV accelerator (Maggiorella et al., 2012). It was reported that silver nanoparticles also had enhancement of the radiation dose deposit in hypoxic glioma cells.

### 2.3 Designing nanoparticles that enhance cancer cells’ sensitivity to radiation by chemical therapeutics

The most common form of radiosensitizers in clinical practice is currently still chemotherapeutic drugs. Corresponding drugs serve as radiosensitizers because of their property by which they lead to the G2-phase cell-cycle arrest, the most radiosensitive section of the cell cycle, and are more prone to inducing cell apoptosis (Nabell and Spencer, 2003). However, a few problems and limitations have been found in clinical practice. Specifically, the non-specific distribution is the primary limit, which influences its therapeutic effect. Moreover, the adverse effects of a cytotoxic agent frequently occur, such as myelosuppression, neurotoxicity, and musculoskeletal toxicity (Markman, 2003). The properties of nanoparticles that are suitable for tumor-targeted drug delivery based on the enhanced permeability and retention (EPR) effect and the active target strategy enable its development as the carrier of chemical drugs. Preferential accumulation in tumors makes it possible to enhance radiation-induced cytotoxicity capability in tumor sites, and poor penetration in nearby normal tissues could reduce the toxicity of normal tissues (Cui et al., 2014). Therefore, the nanoparticles that simultaneously possess chemical medicines and a tumor-target modification showed great promise for enhanced radiosensitization. Michael E *et al*. prepared the formulation of docetaxel polymeric nanoparticles modified by folate (FT-NP Dtxl). When mice were irradiated for 12 h following systemic therapy with FT-NP Dtxl, it revealed the most serious tumor growth inhibition (day 18 compared with 48 h treatment, *p* = 0.03). Furthermore, tumors concurrently treated with FT-NP Dtxl and irradiated for 12 h following systemic therapy consistently exhibited much more dead cells, demonstrated by an increased quantity of condensed nuclei in cells, compared with that of Dtxl. Furthermore, chemical radiosensitizers could be used for targeted delivery to cancer cells through specific ligand modification, leading to targeted radiation sensitization. The combination of PLGA nanoparticles containing a 8-dibenzothiophen-4-yl-2-morpholin-4-yl-chromen-4-one (NU7441) radiosensitizer conjugated with prostate cancer cell-penetrating peptide (R11) along with a suitable imaging agent was prepared for a prostate cancer-specific active targeting. Owing to R11, specifically the combination with the laminin receptor on prostate cancer cells, it results in relatively higher absorption of the nanoparticles by the tumor cells compared with other normal cells in the body (Menon et al., 2015).

### 2.4 Designing antisense oligonucleotide genetic loaded nanoparticles

Contrary to previous attempts to improve the outcome of radiotherapy through the utilization of pharmacological radiosensitizers, gene radiosensitization and the simultaneous dose are not based on the presumption in the minority cell group of oxygen deficit which is the major barrier for radiocurability. Smart nanoparticles carrying antisense oligonucleotides are able to directly target nucleotide homologies. The strategy exhibits more specific genes whose overexpression or inhibition may lead to improved radiosensitivity of tumor cells. Genetic radiosensitization is intended to make all tumor cells with common genetic or phenotypic characteristics sensitive. Currently, antisense Ataxia-telangiectasia mutated (ATM) and the epidermal growth factor receptor (EGFR) genetic therapy had been explored as the radiosensitization strategy on a human cancer cell line (Fan et al., 2000; Guha et al., 2000; ping et al., 2010).

ATM is a large 370-kDa nuclear phosphoprotein comprising the COOH-terminal domain with about 400 residues and is homologous with the catalytic subunits of phosphatidylinositol 3-kinases (Savitsky et al., 1995). Recent studies indicate that ATM can activate the vital regulatory factor of various signal transduction pathways and mediate effective guidance of the signal networks, which are in charge of the cell cycle arrest and the repair of DNA damage induced by ionizing radiation (IR), causing cell recovery and survival after exposure to IR (Rotman and Shiloh, 1999). Consequently, the absence of normal ATM functioning among the hereditary AT syndrome demonstrates that it leads to pleiotropic clinical syndrome related to the apparently growing risk of cancer and serious allergy to ionizing radiation (Sarkaria and Eshleman, 2001). An experimental study of PLGA nanoparticles comprising ATM antisense oligonucleotides (ASOs) was investigated for the radiosensitization of squamous cell carcinoma at the head and neck of mice. Nanoparticles as delivery carriers of sequestering oligonucleotides can protect it from nuclease hydrolysis. The apparently higher radiosensitivity was shown when cells were treated with nanoparticles comprising ATM ASOs and irradiation than when cells were exposed to irradiation only. Simultaneously, the preponderance of radiosensitivity was demonstrated by the lower cloning efficiency and the lower surviving fraction after 2 Gy of irradiation (SF2). Furthermore, the effects of responses to irradiation by nanoparticles containing ATM ASOs *in vivo* were also investigated. The tumor volume of the group treated with nanoparticles comprising ATM ASOs and irradiation was smaller than that of irradiation alone (*p* < 0.05) (Zou et al., 2009). Therefore, nanoparticles as drug delivery carriers for antisense ATM therapy might afford a beneficial therapy when undergoing radiotherapy.

EGFR protooncogene regulates a variety of cell physiological processes, including differentiation, motility, proliferation, survival, blood vessel formation, and DNA repair following genotoxic damage (Schmidt-Ullrich et al., 2003). EGFR showed characteristics of overexpression or over-activation in multiple tumors including head and neck squamous cell carcinoma. Anti-EGFR treatments with irradiation *in vitro* could cause a marked delay in the process of tumor growth and increase radiosensitivity. PLGA nanoparticles-encapsulated antisense EGFR oligonucleotides were used. The SCCVII cells showed great resistance to anti-EGFR nanoparticles or radiation treatment alone; however, there was a co-suppression effect when both treatments were applied together. In addition, antisense EGFR nanoparticles could downregulate the expression of EGFR resulting in the cell cycle blockage in the G1 phase and downregulation of the S phase and also increase the sensitivity of SCCVII cell lines to radiotherapy. ping et al. (2010) Therefore, genetic therapy based on EGFR was also a powerful tool as a radiosensitizer.

### 2.5 Designing nanoparticles that use a unique radiation-activable property

To improve treatment outcomes, chemotherapeutics can be administered during radiotherapy. However, it has some advantages; chemoradiotherapy is in connection with causing increased systemic toxicities, which regularly give rise to the delay or interruption of chemotherapy or radiotherapy (Restivo et al., 2020). It is greatly promising that the smart prodrug may be activated inside tumors through external beam radiation and subsequently enhance radiotherapy, avoiding giving rise to unexpected side effects for normal tissues. Herein, 7-dehydrocholesterol (7-DHC) encapsulated into poly (lactic-co-glycolic acid) nanoparticles was explored as the radiosensitizing agent. Because of its ability to react to radiation-induced ROS and promote radical chain oxidation, the radicalization of 7-DHC is controlled by radiation, which is consistently applicable to tumors during radiation treatment. Furthermore, 7-dehydrocholesterol reductase (DHCR7) has the function for the majority of cancer patients and can turn 7-DHC into non-toxic cholesterol outside the tumors (Prabhu et al., 2016). Compared to cells treated with ionizing radiation (IR) alone, 7-DHC@PLGA NPs associated with IR-treated cells demonstrated an increase of caspase-3 activity of 24.3% and a decreased count of all colonies formed under test radiation doses. In addition, the cell viability percentage at the dosage of two was 0.209 for 7-DHC@PLGA NPs combined with IR, whereas it was 0.530 for a single IR representing the dose-modifying factor of 2.536 (Delahunty et al., 2022). Gao *et al*. synthesized a maytansinoid prodrug, nitrosylated maytansinoid (DM1-NO), and loaded onto poly (lactide-co-glycolic)-block-poly (ethylene glycol) (PLGA-b-PEG) nanoparticles. Interestingly, PLGA enclosure and S-nitrosylation had been shown to inhibit the toxicity of DM1. The aforementioned smart nanoparticles allowed for targeted delivery of curative drugs to tumors through the EPR effect. The following radiation elevated oxidative stress in tumor cells, inducing the break of the S-N chemical bond and the release of DM1 and NO. Both the aforementioned molecules are powerful radiosensitizers. In fact, through studies of the therapeutic effect on the H1299 tumor-bearing nude mice *in vivo*, an elevation of about 9.64 times in the tumor inhibition rate (TIR) for DM1-NO-NPs and RT in comparison with RT only had been demonstrated, and the TIR for DM1-NO-NPs + RT was much higher than that for DM1-NO + RT and DM1-NPs + RT. The low toxicity of the prodrug loaded in nanoparticles was performed in animals through the levels of blood cells, red blood cells, platelets, plateletcrit, alanine transaminase, aspartate transaminase, bilirubin, and creatinine. Therefore, the significance of improving the radiotherapeutic effect along with radiation-activable radiosensitizers cannot be overemphasized.

## 3 Challenges and opportunities in nanoparticle-based radiosensitizers for RT

The specific accumulation of nanoparticle-based radiosensitizers in a tumor is the premise of targeted delivery *in vivo*. It is mainly achieved through passive targeting and active targeting. The realization of active targeting is relied upon for the recognition of ligands loaded with tumor-marker molecules in smart nanoparticles. Since nanoparticle-based drug delivery systems orientating tumor cells by passive targeting heavily depend on the EPR effect, it remains the bottleneck in using this approach for advanced nano-targeting therapies. It has become increasingly recognized when there is large individual heterogeneity in EPR-based tumor targeting. The disparity examined in tumor accumulation may be partly attributed to the commonly found variety of the angiogenic activity that correlates to EPR activity and tumor aggressivity (Choi et al., 2015) since that is quite different from human tumors as well (Karathanasis et al., 2009). Although the EPR effect is a fundamental principle of tumor targeting, its heterogeneity among human tumors is the main disadvantage to the successful clinical experience of macromolecular drugs and a delivery system. In addition, although animal tumors are able to grow within a few weeks to establish an application model, human tumors are formed after a much longer period of time and often display evident intratumoral heterogeneity with regard to genetic and physiological changes which cannot be detected in a rapidly forming animal model (Heppner, 1984). Innovative approaches to these drawbacks are urgently needed to achieve more remarkable and effective drug delivery for antitumor therapy. Furthermore, nanoparticles also tend to accumulate in the normal organs of the body, especially in the liver and kidneys. For radiotherapy, the dose is a critical issue because it will not only destroy the cancer cells but also damage the surrounding healthy cells at a higher dose. Therefore, further study is needed to realize a safe and efficient radiosensitizer based on nanoparticles being successfully applied in clinical settings.

Despite these strategies of radiosensitizers, higher generation of reactive oxygen species, and utilization of high-atomic-number nanoparticles (used in genetics or chemotherapeutics), different studies have shown the radiosensitizing effect of smart nanoparticles; however, the length of the persistence of the effects and the best interval between radiosensitizers and radiation remain unclear. Evidence suggests that the synergistic effect of cisplatin combined with radiation may rely on the combination protocol and the tumor type (Wang et al., 2002). In the future, it will be meaningful to deeply analyze the cytotoxicity impact of the smart nanoparticle-radiation sequence and exposure time on specific tumors, and, depending on the investigation, determine optimal conditions for radiation.

Fortunately, the potential new radiosensitizer NBTXR3, jointly developed by Nanobiotix and LianBio, has been registered and launched in an international phase III trial in China. It is carried out to evaluate the survival outcome of NBTXR3 selected by the researchers for radiotherapy alone or radiotherapy-activated intratumoral injection with cetuximab in elderly patients presenting with locally advanced head and neck squamous cell carcinoma who are not suitable for chemotherapy with a platinum-containing drug regimen. NBTXR3 is composed of functionalized hafnium dioxide (HfO_2_) nanoparticles. All in all, the ideal smart nanoparticles as a potential tool for radiation dose enhancement have properties that are tumor targeting accompanied by marginal systemic toxicity, providing improved radiation efficacy with reduced energy delivery, while reducing the side effects of healthy tissues surrounding the tumors.
